# The Validity of the ROX Index and APACHE II in Predicting Early, Late, and Non-Responses to Non-Invasive Ventilation in Patients with COVID-19 in a Low-Resource Setting

**DOI:** 10.3390/v15112231

**Published:** 2023-11-08

**Authors:** Sumalatha Arunachala, Ashwaghosha Parthasarathi, Chetak Kadabasal Basavaraj, Mohammed Kaleem Ullah, Shreya Chandran, Hariharan Venkataraman, Prashant Vishwanath, Koustav Ganguly, Swapna Upadhyay, Padukudru Anand Mahesh

**Affiliations:** 1 Department of Respiratory Medicine, JSS Medical College, JSS Academy of Higher Education and Research, Mysuru 570015, India; a.suma86@gmail.com (S.A.); drchetak@gmail.com (C.K.B.); shreyachandran@gmail.com (S.C.); harihar5597@gmail.com (H.V.); 2 Public Health Research Institute of India, Mysuru 570020, India; 3 Department of Critical Care Medicine, Adichunchanagiri Institute of Medical Sciences, Bellur 571448, India; 4 Allergy, Asthma, and Chest Centre, Krishnamurthypuram, Mysuru 570004, India; ap2320@rwjms.rutgers.edu; 5 Rutgers Centre for Pharmacoepidemiology and Treatment Science, New Brunswick, NJ 08901, USA; 6 Centre for Excellence in Molecular Biology and Regenerative Medicine (A DST-FIST Supported Center), Department of Biochemistry (A DST-FIST Supported Department), JSS Medical College, JSS Academy of Higher Education and Research, Mysore 570015, India; ka7eem@jssuni.edu.in (M.K.U.); prashantv@jssuni.edu.in (P.V.); 7 Division of Infectious Disease and Vaccinology, School of Public Health, University of California, Berkeley, CA 94720, USA; 8 Unit of Integrative Toxicology, Institute of Environmental Medicine (IMM), Karolinska Institutet, 17177 Stockholm, Sweden; koustav.ganguly@ki.se

**Keywords:** COVID-19, ROX index, high-flow nasal oxygenation, HFNO, non-invasive ventilation, NIV, APACHE II, intensive care unit

## Abstract

The use of the Ratio of Oxygen Saturation (ROX) index to predict the success of high-flow nasal oxygenation (HFNO) is well established. The ROX can also predict the need for intubation, mortality, and is easier to calculate compared with APACHE II. In this prospective study, the primary aim is to compare the ROX (easily administered in resource limited setting) to APACHE II for clinically relevant outcomes such as mortality and the need for intubation. Our secondary aim was to identify thresholds for the ROX index in predicting outcomes such as the length of ICU stay and failure of non-invasive respiratory support therapies and to assess the effectiveness of using the ROX (day 1 at admission, day 2, and day 3) versus Acute physiology and chronic health evaluation (APACHE) II scores (at admission) in patients with Coronavirus Disease 2019 (COVID-19) pneumonia and Acute Respiratory Distress Syndrome (ARDS) to predict early, late, and non-responders. After screening 208 intensive care unit patients, a total of 118 COVID-19 patients were enrolled, who were categorized into early (n = 38), late (n = 34), and non-responders (n = 46). Multinomial logistic regression, receiver operating characteristic (ROC), Multivariate Cox regression, and Kaplan–Meier analysis were conducted. Multinomial logistic regressions between late and early responders and between non- and early responders were associated with reduced risk of treatment failures. ROC analysis for early vs. late responders showed that APACHE II on admission had the largest area under the curve (0.847), followed by the ROX index on admission (0.843). For responders vs. non-responders, we found that the ROX index on admission had a slightly better AUC than APACHE II on admission (0.759 vs. 0.751). A higher ROX index on admission [HR (95% CI): 0.29 (0.13–0.52)] and on day 2 [HR (95% CI): 0.55 (0.34–0.89)] were associated with a reduced risk of treatment failure. The ROX index can be used as an independent predictor of early response and mortality outcomes to HFNO and NIV in COVID-19 pneumonia, especially in low-resource settings, and is non-inferior to APACHE II.

## 1. Introduction

In COVID-19 patients, the severity of the illness can be assessed using scoring systems like APACHE II and SOFA based on clinical parameters such as vital signs and laboratory values [1]. However, their use can be challenging in low- and middle-income countries due to a lack of skilled personnel and electronic ICU systems [2]. COVID-19 has worsened this issue and delays in treatment may result. Simpler systems like neutrophil-to-lymphocyte ratio (NLR) may be useful in predicting disease severity, but scoring systems predicting responses to therapy are also needed. The Ratio of Oxygen Saturation (ROX) index is used in the initial assessment of COVID-19 patients and triaging in emergency departments [3]. It is also used to predict the deterioration of hospitalized COVID-19 patients in the wards [4] and for predicting the need for early invasive mechanical ventilation [5]. It is widely accepted for monitoring patients using HFNO. Thus, the ROX index has the potential to predict the failure of therapies such as HFNO and NIV in severe COVID-19 patients admitted to the ICU with Acute Respiratory Distress Syndrome (ARDS).

To date, there has been no research into whether the ROX index can be used serially to anticipate and categorize COVID-19 patients into early, late, and non-responders prior to the onset of respiratory failure that requires invasive mechanical ventilation. This is especially important during a pandemic, as early identification and appropriate allocation of resources for these patients is crucial. The ROX index, which includes simple parameters like respiratory rate, SpO_2_, and FiO_2_, can be monitored by emergency nurses to predict the need for intubation [6]. If clinicians can predict the deterioration of patients early using such indices, it would prompt early treatment decisions using simple algorithms and better allocation of resources, such as the need for ventilators or ECMO, for these patients [7]. 

In this prospective study, the primary aim is to compare the ROX (easily administered in a resource-limited setting) with APACHE II for their clinically relevant outcomes such as mortality and the need for intubation. Our secondary aim was to identify thresholds for the ROX index in predicting outcomes such as the length of ICU stay and failure of non-invasive respiratory support therapies, and to assess the effectiveness of using the ROX (day 1 at admission, day 2, and day 3) and the Acute physiology and chronic health evaluation (APACHE) II scores (at admission) in patients with Coronavirus Disease 2019 (COVID-19) pneumonia and Acute Respiratory Distress Syndrome (ARDS) to predict early, late, and non-responders.

## 2. Materials and Methods

This is a prospective cohort study conducted on patients admitted to a tertiary care university teaching hospital, Mysore, from September 2020 to November 2020 after obtaining clearance from the institutional ethics committee (JSSMC/IEC/141020/09 NCT)/2020-21). Information was collected from all patients who tested positive for COVID-19 RT-PCR, required ICU care, and needed NIV or HFNO were eligible to be included in the study. Criteria for ICU admission was based on Infectious Disease Society of America (IDSA)/American Thoracic Society (ATS) guidelines on management of severe community-acquired pneumonia [8] and included patients fulfilling one major or 2 minor criteria as follows: Major criteria—need for mechanical ventilation and septic shock with the need for vasopressors; minor criteria—respiratory rate > 30 breaths per minute, PaO_2_/FiO_2_ < 250, confusion, disorientation, leukopenia (<4000 cells/cubic millimeter), thrombocytopenia (<1 lakh cells/cubic millimeter), uremia (Blood urea nitrogen > 20 mg/dL), multilobar infiltrates, hypotension requiring aggressive fluid resuscitation, and hypothermia. Acute Hypoxic Respiratory Failure was defined as PaO_2_ < 60 mmHg and PaCO_2_ < 45 mm Hg, presence of dyspnea, and RR > 35 cycles/minute after excluding renal and cardiac causes of breathlessness. Those who required oxygenation and mechanical ventilation upon ICU admission, Glasgow coma scale < 12, palliative care, and were discharged against medical advice were excluded.

Information collected included patient’s demographics characteristics, comorbidities, vitals, laboratory tests, hematological parameters, APACHE II (Acute physiology and chronic health evaluation) score on admission, presence or absence of MODS, and septic shock. Serial ROX was calculated within an hour after admission (day 1), at the beginning of day 2, and at the beginning of day 3. ROX index was calculated irrespective of the type of non-invasive ventilatory support.

Treatment administered included steroids (inj Dexamethasone 6 mg OD), anticoagulants (inj heparin 5000 units iv BD), and antibiotics (as per ICU protocol) in all patients. Six patients received immunomodulators (inj tocilizumab 6 mg/kg infusion over 6 h). All patients received treatment for COVID-19, which was standardized across all patients according to the National Institute of Health’s COVID-19 guidelines [9]. Awake proning was encouraged irrespective of the type of non-invasive respiratory support. However, the compliance and duration has not been measured. As per the ICU protocol, temperature was monitored by nurses every 6 h and any temperature > 101 °C was considered fever. It was treated with intravenous paracetamol.

Indications for NIV included RR > 25 cycles per minute and/or saturation of less than 92% while breathing on oxygen of 10 L/min via a face mask (an approximate FiO_2_ of 60%) as per the National Institute of Health’s COVID-19 guidelines [9]. Indications for HFNO included hypoxemia on 10 L of oxygen (SpO_2_ < 92%) via face mask, RR > 25 cycles per minute, and contra-indications to NIV such as severe facial deformity and facial burns.

### 2.1. Settings for HFNO

High-Flow Nasal Oxygen (HFNO) therapy involved adjusting the flow rate between 30 and 60 L/min based on the patient’s condition and maintaining a temperature range of 34–37 °C. The fraction of inspired oxygen (FiO_2_) was regulated to keep the peripheral blood oxygen saturation (SpO_2_) between 88 and 92%. Vital signs and arterial blood gases were closely monitored.

### 2.2. Settings for NIV

For Non-Invasive Ventilation (NIV), a ventilator was used with an appropriately sized face mask. The mode employed pressure support with positive end-expiratory pressure, starting with an initial inspiratory pressure of 8–10 cm H_2_O to achieve a tidal volume of 6 mL/kg body weight. Positive end-expiratory pressure was set at a minimum of 5 cm H_2_O and adjusted based on the patient’s response. FiO_2_ was also adjusted to maintain the target SpO_2_ of 88–92%. The settings were continuously adapted according to the patient’s clinical response.

Switching from NIV to HFNO or otherwise was as per the discretion of the treating clinician. Switching was allowed for the following reasons: lack of patient cooperation, patient discomfort or refusal, non-compliance with proning, and/or requirement of higher PEEP (for switch to NIV). Switching was not permitted if there was a defined failure of NIV/HFNO. In such cases, invasive mechanical ventilation was instituted. Combination therapy with both HFNO and NIV was permitted and ROX index was calculated irrespective of the type of non-invasive respiratory support, since the primary aim was to compare ROX index with APACHE II.

### 2.3. Criteria for Failure of Response to HFNO [10]

- Oxygenation criteria: SpO_2_ < 88% for more than 6 h while receiving HFNO with a flow of 60 L/min and partial pressure of arterial oxygen/fraction of inspired oxygen (P/F) ratio < 100.
- Ventilation criteria: presence of respiratory acidosis with a pH < 7.25.
- Work of breathing criteria: tachypnoea with RR > 30 and the use of accessory muscles of respiration.
- Others: need for invasive mechanical ventilation due to hemodynamic instability despite fluid resuscitation (systolic blood pressure < 90 mm of Hg and/or mean arterial pressure less than 65 mm of Hg), altered sensorium (GCS < 12), need for airway protection, and dysrhythmias causing hemodynamic instability and cardiopulmonary arrest.

### 2.4. Criteria for Failure of Response to NIV [11]

- Oxygenation criteria: SpO_2_ < 88% for more than 6 h while receiving NIV with a P/F ratio < 100, and a minimum PEEP of 5 cm of water and minimum pressure support of 5 cm of water.
- Ventilation criteria: presence of respiratory acidosis with a pH < 7.25.
- Work of breathing criteria: tachypnoea with RR > 30 and the use of accessory muscles of respiration.
- Others: need for invasive mechanical ventilation due to hemodynamic instability despite fluid resuscitation (systolic blood pressure < 90 mm of Hg and/or mean arterial pressure less than 65 mm of Hg), altered sensorium (GCS < 9), need for airway protection, dysrhythmias causing hemodynamic instability, and cardiopulmonary arrest.

The pandemic overwhelmed the surge capacity of our ICU. There was limited accessibility of invasive mechanical ventilation, and outcomes on invasive mechanical ventilation were extremely poor (100% mortality in initial one month of the pandemic). As the pandemic progressed, many of the nurses and doctors were affected by COVID-19, further reducing the staff to patient ratio. Hence, a local ICU policy was made to extend the trial of non-invasive respiratory support to 6 h. We defined HFNO/NIV failure as per this policy. In the event of HFNO or NIV failure, patients were treated with invasive mechanical ventilation.

We have arbitrarily categorized the patients into 3 groups based on the time taken to respond to therapy and the outcome.

- Early responders—responded well within 5 days and weaned off HFNO/NIV, discharged after complete recovery.
- Late responders—response time more than 5 days and weaned off HFNO/NIV, shifted to general wards after completing ICU stay.
- Non-responders—patients who failed NIV and/or HFNO and required invasive mechanical ventilation at any time after hospital admission or patients who succumbed to illness.

Patients were shifted out of the ICU once the following criteria were met: patient receiving < 10 L of oxygen support for >24 h, hemodynamic stability, absence of worsening renal failure or liver failure, absence of dyselectrolytemia, presence of stable neurological status, and ability to protect the airway.

### 2.5. Statistical Analysis

Statistical analysis was performed using Jamovi (v1.6, The Jamovi project, SYD, AUS). Data were tested for normality using the Shapiro–Wilk test. Continuous variables were presented as mean ± standard deviation if they were normally distributed or median with their interquartile range if not normally distributed. Categorical variables were presented as percentages. Statistical significance was assessed by Student’s *t*-test or Wilcoxon’s test for continuous variables depending on the normality of the distribution of data. For categorical variables, Pearson’s chi-square test was employed.

The area under the curve (AUC), sensitivity, specificity, and optimal cut-off values (determined by Youden’s index) were calculated based on the receiver operating characteristic (ROC) curve. Multivariable analyses were conducted to identify independent variables associated with early and late responses to non-invasive ventilatory support. Variables presumed to be of clinical importance were included in the model. Along with the aforementioned variables, those with a significant association with the response (found using a simple chi-square test or Student’s *t*-test) were added to the model. The hazard ratio (HR) was calculated using Cox proportional hazards regression analysis. The 30-day survival curves were created using the Kaplan–Meier method, and the survival rates were compared using the log-rank test. A two-tailed *p*-value of <0.05 was considered statistically significant.

## 3. Results

**Figure 1:**
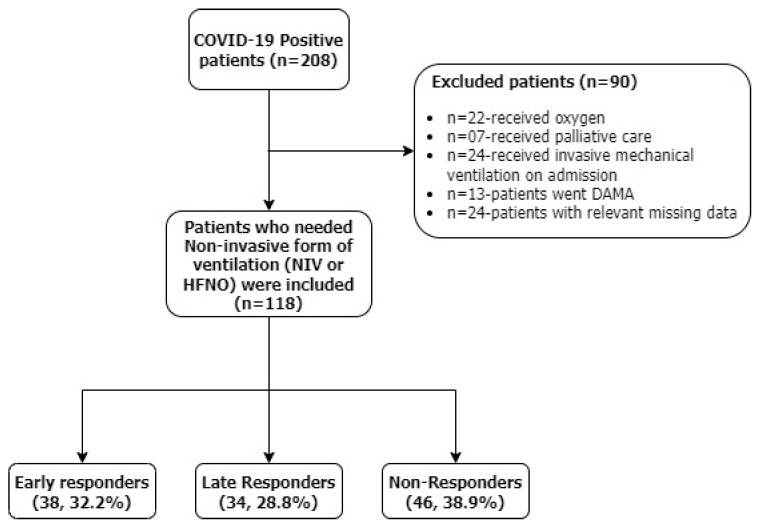
Flowchart illustrating the distribution of the study population.

A total of 208 patients with RT-PCR confirmed diagnosis of COVID-19, out of which 118 patients who needed non-invasive ventilation were enrolled in the study, of which 38 were early responders, 34 were late responders, and 46 were non-responders (Figure 1).

There was no significant difference in age and sex (male preponderance in all three groups (71.1% vs. 85.3% vs. 80.4%). The average duration of ICU stay among the three groups for Early vs. Late vs. Non-responders was 7.5 (6.0–9.0) vs. 10.0 (8.0–11.6) vs. 10.0 (8.7–16.0), respectively. Additionally, we found that the respiratory rate of the non-responder group was significantly higher compared with the early and late responder group (Early vs. Late vs. Non-responders: Respiratory rate: 28.0 (26.0–31.7) vs. 32.0 (28.0–40.0) vs. 34.0 (28.0–39.0) *p* = 0.01), respectively. The other vital signs showed no statistically significant differences between the groups (Table 1).

Upon comparison of hematological parameters between the three groups, we found that the non-responders had a significantly higher serum AST (Early vs. Late vs. Non-responders: 32.0 (26.0–45.8) vs. 33.0 (24.2–53.8) vs. 59.0 (43.3–81.3), respectively; *p* < 0.01), urea (Early vs. Late vs. Non-responders: 28.0 (20.2–37.8) vs. 38.0 (31.0–49.0) vs. 40.0 (31.3–62.8); *p* < 0.01), creatinine (Early vs. Late vs. Non-responders 0.8 (0.7–1.0) vs. 0.9 (0.7–0.9) vs. 1.1 (0.8–1.4); *p =* 0.01), and blood urea nitrogen levels (Early vs. Late vs. Non-responders: 13.1 (9.4–17.7) vs. 17.7 (14.5–22.9) vs. 18.7 (14.6–29.3); *p* < 0.01) (Table 1). Furthermore, we found a significantly higher prevalence of complications in the non-responder group compared with the early and late responders. Non-responders (26.1%) had significantly higher chronic cardiac disease compared with early (10.5%) and late responders (8.8%). However, there was no other significant difference in the prevalence of comorbidities between the groups. Interestingly, APACHE II scores on admission were significantly higher (Early vs. Late vs. Non-responders: 9.0 (5.4–11.6) vs. 11.0 (8.4–14.6) vs. 15.0 (10.0–18.3), respectively; *p* < 0.01) in the non-responders. The ROX index scores on admission (within 1 h, day 1) (Early vs. Late vs. Non-responders: 6.4 (5.5–7.7) vs. 4.3 (3.5–5.8) vs. 4.1 (3.7–5.0), respectively; *p* < 0.01), at the beginning of day 2 (Early vs. Late vs. Non-responders: 7.4 (5.3–9.2) vs. 4.9 (4.1–5.8) vs. 4.4 (3.6–5.7); *p* < 0.01), and at the beginning of day 3 (Early vs. Late vs. non-responder: 7.8 (6.2–9.9) vs. 5.6 (4.2–7.1) vs. 4.2 (3.3–5.8); *p* < 0.01) were significantly lower in the non-responder group compared with the early and late responders (Table 1). The serial ROX measurements in each of the three groups were evaluated using repeated measures ANOVA, which showed a statistically significant increase in early (*p* = 0.035) and late responders (*p* = 0.028) and no significant change in non-responders (*p* = 0.69).

Multinomial logistic regression of late vs. early responders showed that the higher ROX index scores on admission [OR (95% CI): 0.468 (0.2939–0.745)], on day 2 [OR (95% CI): 0.599 (0.412–0.872), and on day 3 [OR (95% CI): 0.552 (0.358–0.851) were associated with a reduced risk of treatment failure. Multinomial logistic regression of the non- vs. early responders groups showed that the ROX index scores on admission [OR (95% CI): 0.39 (0.23–0.663)], on day 2 [OR (95% CI): 0.472 (0.306–0.729)], and on day 3 [OR (95% CI): 0.502 (0.307–0.82)] were associated with a reduced risk of treatment failure. APACHE II scores [OR (95% CI): 1.216 (1.1024–1.34)], sepsis [OR (95% CI): 8.365 (1.3956–50.14)], and chronic cardiac disease [OR (95% CI): 3.829 (1.0252–14.3)] were associated with an increased risk of treatment failure (Table 2).

ROC analysis for early vs. late responders observed that APACHE II scores on admission had the largest area under the curve (0.847) with a sensitivity of 84.62% and specificity of 68.35%. This was followed by the ROX index on admission that had an AUC of 0.843 (Table 3). For responders vs. non-responders, we found that the ROX index on admission (SENS: 73.21%; SPE: 72%) had a slightly better AUC than APACHE II (SENS: 65.28%; SPE: 76.09%) on admission (0.759 vs. 0.751) (Table 4).

Univariable Cox proportional hazards regression analysis revealed that APACHE II scores on admission as well as complications like chronic cardiac disease, sepsis, Multiple Organ Dysfunction Syndrome (MODS), and acute kidney injury (AKI) were significant risk factors for the prediction of intubation. On multivariable logistic regression analysis, we found that APACHE II [HR (95% CI): 1.12 (1.03–1.21)] as well as chronic cardiac disease [HR (95% CI): 1.09 (1.02–1.21)], sepsis [HR (95% CI): 5.87 (1.27–45.18)], MODS [HR (95% CI): 7.89 (2.12–40.58)], and AKI [HR (95% CI): 5.80 (1.63–28.22)] were independent risk factors for treatment failure. Our observations indicate that a higher ROX index on admission [Multivariable HR (95% CI): 0.29 (0.13–0.52)] and on day 2 [HR (95% CI): 0.55 (0.34–0.89) were associated with a reduced risk of treatment failure (Table 5).

**Figure 2:**
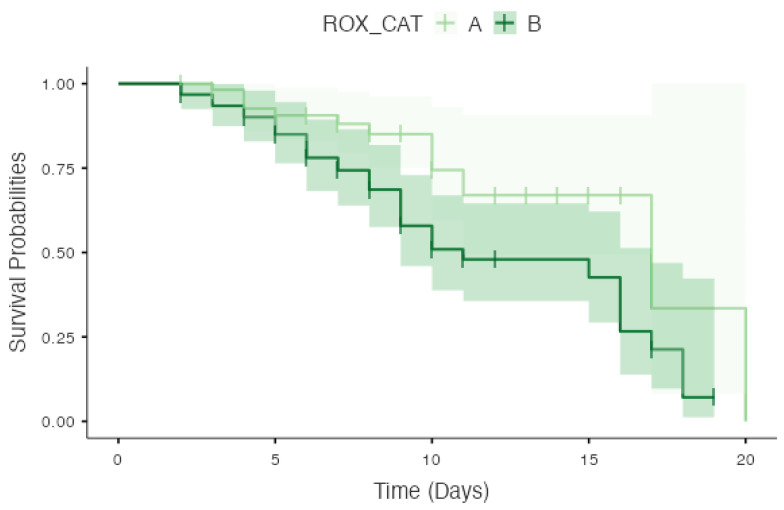
Kaplan–Meier survival curve showing survival probabilities for low and high ROX index scores. ROX_CAT A = ROX score < 3.1; ROX_CAT B = ROX score > 3.1.

A significant difference in survival possibilities was observed between high and low ROX cut-off scores (cut-off: 3.1) following Kaplan–Meier analysis (log-rank test; *p* = 0.021) (Figure 2).

## 4. Discussion

In our study, we have introduced a novel concept of categorizing patients into early, late, and non-responders according to the time taken to respond to therapy in COVID-19 patients admitted to the ICU. There was significant improvement in the serial ROX indices over 48 h among early and late responders but not in non-responders. The findings of our study show that the ROX at admission with a cut-off of >5.2 (Table 3) predicts early response to non-invasive therapies of respiratory support, whereas a cut-off of <4 (Table 4) suggests a need for invasive mechanical ventilation and a cut-off of <3.1 (Figure 2) increases the risk of mortality in COVID-19 patients.

The ROX is a simpler and less time-consuming alternative to APACHE II for predicting response to therapy in COVID-19 patients. In LMICs, calculating daily APACHE II is challenging due to lower doctor-to-patient ratios, lack of electronic ICUs, and additional laboratory data requirements, which can be financially burdensome for patients. Our study is the first to compare APACHE II with the ROX in COVID-19 patients and finds that the ROX index is comparable to APACHE II in identifying respiratory failure and the need for invasive mechanical ventilatory support. Additionally, the ROX may be superior in predicting the response to therapy. Therefore, clinicians in LMIC countries may consider baseline APACHE II and serially calculate the ROX to monitor COVID-19 patients in the ICU.

The ROX index, calculated as the ratio of pulse oximetry to the fraction of inspired oxygen (SpO_2_/FiO_2_) over respiratory rate (RR), serves as a valuable tool in emergency and critical care settings, facilitating prompt decisions on various aspects of clinical decision making, thereby enhancing patient care [12]. Its ease of assessment, simplicity, objective assessment (eliminating subjective bias), and repeatability make it a valuable tool for assessment even by non-healthcare personnel [13]. This underscores the pivotal role of the ROX index, streamlining decision-making processes for clinicians and enabling early triaging, early prognostication regarding the probable disease trajectory, mechanical ventilation decision support, risk stratification, and resource management [13]. Also, the ROX index is sensitive to changes in patient’s breathing mechanics due to various other non- respiratory causes. It is affected by pain, acidosis, fever, hypotension, and immobilization [12]. Hence, serial measurement of the ROX is better over a single static measurement as small variations in its components (for example, respiratory rate) may produce very diverse scores. Recently, the ROX index has also been evaluated for predicting mortality. Basoulis et al. evaluated the serial ROX indices at 12 h on days 2, 3, and 7 for the prediction of HFNO failure and mortality [14]. They found an ROX index of <4.4 measured at 12 h and a predictor of mortality similar to the findings of our study. They also suggested use of the ROX index as a daily assessment tool, as they found significant improvement in the ROX values among the success group and the absence of such improvement in the failure group, similar to the findings of our study. Leszek et al. evaluated the ability of the median ROX index (between zero hours and 8 h) to predict survival among intubated COVID-19 patients [15]. They found that a cut-off >7 was best to predict survival. Thus, the ROX index can also predict mortality in COVID-19 patients.

Our study has proposed a fresh approach to categorize COVID-19 patients in the ICU as early, late, or non-responders depending on the duration of their response to treatment. The results of our research indicate that an ROX index score greater than 5.2 can predict an early response to non-invasive respiratory support therapies. This method of categorization was previously employed by Blasi et al. in their study of community-acquired pneumonia patients, where they used Halm’s criteria to evaluate the time it took for 2039 patients to exhibit a clinical response [16]. In patients with an early response to therapy (median duration of time to clinical response was 3 days compared with 7 days in late responders), they reported a decreased length of ICU stay and lower ICU resource use. Predicting ICU outcomes is crucial for effective allocation of resources and timely interventions to improve patient outcomes [17,18]. Early identification of response to treatment can prevent complications and facilitate referrals for palliative care [19]. Scoring systems like APACHE II, SAPS, and SOFA are commonly used, but simpler indices such as NLR and the ROX have gained popularity during the COVID-19 pandemic [19,20].

Our study indicates that in patients with an ROX index score less than 4 measured at admission, it predicts the need for invasive mechanical ventilation. Other studies have also shown that an ROX score of less than 3.5 at various time points is a predictor of failure of high-flow nasal oxygen therapy. Blez et al. found that an ROX index score of greater than 4.88 at the start of high-flow nasal oxygen therapy had a sensitivity of 81% and specificity of 38% in detecting treatment success [21], while Calligaro et al. found an ROX index of >3.6 at 6 h after initiation of HFNO predicted a successful outcome [22]. A similar study by Chandel et al. found a cut-off of ROX > 3.6 to predict HFNO success [23], whereas Panadero et al. found that an ROX of <4.94 predicted HFNO failure [24]. Colaianni-Alfonso et al. evaluated combination therapy with HFNO and CPAP for moderate COVID-19 ARDS and found that the ROX index successfully predicted the failure of combined HFNO and CPAP therapies [25]. With the ROX index of 6.28 at 12 h as the cut-off value to predict failure (intended as IMV), the sensitivity was 97.6% and specificity was 51.8%. Our study observed that an ROX index of 4 or less at admission predicted failure with a sensitivity of 73.2% and specificity of 72%. The differences in the ROX index observed could be due to the fact that the ROX index was measured at different time points and included patients of varying severity.

In all these studies, the reason for identification of the ROX threshold could be due to the fact that the ROX index was measured at different time points (baseline, 6, 12, and 24 h), included patients of varying severity, and used different devices (CPAP, HFNO, and Ventilator NIV). A useful clinical cut-off was identified by the recent systematic review and meta-analysis that included 1301 patients with COVID-19 pneumonia treated with HFNO and found an ROX of <5 to be a predictor of need for invasive mechanical ventilation [26].

Numerous studies have examined factors associated with mortality in critically ill COVID-19 patients. Studies from Sweden, Kuwait, Spain, and Italy have identified comorbidities like advanced age, hypertension, type 2 diabetes, chronic kidney disease, and the need for invasive mechanical ventilation as risk factors for mortality [27,28,29,30]. A systematic review of 19 studies found that comorbidities, ARDS, and history of smoking were risk factors for mortality [31]. However, our study did not find hypertension, type 2 diabetes, or COPD as risk factors for mortality. Differences in study findings may be due to variations in the duration and control of non-communicable diseases, end-organ involvement, medication use, and racial differences. Studies have also evaluated sepsis, acute kidney injury, ARDS, microvascular dysfunction, and coagulation abnormalities as independent predictors of mortality, with similar findings to our study [32].

To the best of our knowledge, this study is the first of its kind to categorize COVID-19 pneumonia patients as early, late, and non-responders and assess the response to non-invasive respiratory methods using the ROX index. There are certain limitations to our study. This is an observational cohort study from a single center with a relatively small sample size. As we obtained data in a resource-poor setting, we were unable to collect data on serial hourly measurements of the ROX index and blood gas analysis. We also did not collect data on awake-prone positioning, which could have significantly altered the results.

## 5. Conclusions

The ROX index has shown promise as a useful tool for predicting early response, treatment failure, and mortality outcomes in COVID-19 pneumonia patients receiving high-flow nasal oxygen (HFNO) and non-invasive ventilation (NIV), particularly in low-resource settings. This index is comparable to the widely used APACHE II score in terms of its predictive ability. The serial ROX index has the potential to predict early, late, and non-responders. However, further research is needed to confirm these findings and to fully evaluate the clinical utility of the ROX index in managing COVID-19 patients.

## Figures and Tables

**Table 1:** Describes the demographic, clinical, and test of significance between early, late, and non-responder subjects with COVID-19.

	Early Responders (N = 38)	Late Responders (N = 34)	Non-Responders (N = 46)	*p*-Value
Age (years)	59.0 (45.0–67.0)	58.0 (50.9–66.2)	62.0 (52.0–70.1)	0.17 *
Male %	27 (71.1)	29 (85.3)	37 (80.4)	0.32 ^†^
Female %	11 (28.9)	5 (14.7)	9 (19.6%)	
Duration of ICU stay (days)	7.5 (6.0–9.0)	10.0 (8.0–11.6)	10.0 (8.7–16.0)	0.01 *
Mode of Intervention	22 (57.9)	17 (50.0)	33 (71.1)	0.13 ^†^
Vitals				
Heart Rate (beats/minute)	108.0 (94.3–116.0)	100.0 (97.0–117.3)	98.0 (88.2–112.0)	0.28 *
Respiratory Rate	28.0 (26.0–31.7)	32.0 (28.0–40.0)	34.0 (28.0–39.0)	0.01 *
Systolic blood pressure	130.0 (120.0–140.0)	140.0 (120.0–150.0)	140.0 (130.0–148.3)	0.22 *
Diastolic blood pressure	70.0 (70.0–80.0)	80.0 (70.0–80.0)	80.0 (70.0–88.3)	0.24 *
PH	7.5 (7.4–7.5)	7.4 (7.4–7.4)	7.4 (7.4–7.5)	0.10 *
PCO_2_	30.7 (28.9–34.2)	33.8 (27.4–37.4)	30.6 (28.2–34.6)	0.87 *
PaO_2_	78.3 (63.7–89.8)	65.1 (54.0–96.1)	72.7 (57.8–83.9)	0.22 *
P/F ratio	156.6 (122.8–193.6)	98.0 (77.8–169.3)	104.6 (73.0–144.9)	<0.01*
Hematological investigations				
Hemoglobin (g/dL)	13.5 (11.7–14.6)	13.8 (12.8–14.6)	13.3 (11.6–13.9)	0.42 *
WBC count (X1000 cells/cu. mm)	7290.0 (4826.7–9595.0)	8980.0 (7405.0–10,853.3)	11,590.0 (8038.3–13,136.7)	<0.01 *
Absolute Neutrophil count	6699.6 (4204.8–9742.7)	8037.8 (6422.6–9611.6)	10,167.2 (7067.1–11,928.4)	0.01 *
Absolute Lymphocyte count	800.9 (588.4–1208.3)	930.0 (572.1–1182.0)	745.0 (574.9–1111.3)	0.42 *
Platelet count	2.2 (1.9–2.9)	2.2 (1.7–2.8)	2.6 (1.7–3.2)	0.71 *
Procalcitonin	0.3 (0.1–0.7)	0.2 (0.1–0.4)	0.7 (0.2–1.7)	0.03 *
C-Reactive Peptide	108.1 (52.5–172.3)	89.0 (41.0–203.0)	88.8 (41.3–168.7)	0.49 *
Serum Albumin	3.3(2.9–3.6)	3.3 (3.1–3.5)	3.3 (3.1–3.6)	0.72 *
Serum AST	32.0 (26.0–45.8)	33.0 (24.2–53.8)	59.0 (43.3–81.3)	<0.01 *
Serum ALT	28.0 (21.0–36.8)	30.0 (22.2–57.7)	41.0 (23.0–63.5)	0.13 *
Urea	28.0 (20.2–37.8)	38.0 (31.0–49.0)	40.0 (31.3–62.8)	<0.01 *
Creatinine	0.8 (0.7–1.0)	0.9 (0.7–0.9)	1.1 (0.8–1.4)	0.01 *
Blood Urea Nitrogen	13.1 (9.4–17.7)	17.7 (14.5–22.9)	18.7 (14.6–29.3)	<0.01 *
Sodium	135.0 (132.0–138.0)	135.0 (131.2–138.8)	135.0 (131.0–139.0)	0.63 *
Potassium	4.4 (4.0–4.9)	4.3 (3.7–4.4)	4.4 (4.0–4.8)	0.79 *
Comorbidities				
Diabetes Mellitus	23 (60.5)	21 (61.8)	23 (50.0)	0.49 ^†^
Hypertension	20 (52.6)	20 (58.8)	25 (54.3)	0.86 ^†^
Chronic Cardiac disease	4 (10.5)	3 (8.8)	12 (26.1)	0.06 ^†^
Chronic Kidney disease	4 (10.5)	3 (8.8)	2 (4.3)	0.54 ^†^
Chronic respiratory disease	1 (2.6)	2 (5.9)	3 (6.5)	0.70 ^†^
Complications				
Sepsis	2 (5.3)	2 (5.9)	23 (50.0)	<0.01 ^†^
MODS	3 (7.9)	6 (17.6)	31 (67.4)	<0.01 ^†^
Acute Kidney Injury	2 (5.3)	7 (20.6)	23 (50.0)	<0.01 ^†^
Intubation	0 (0)	0 (0)	46 (100)	<0.01 ^†^
Mortality	0 (0)	0 (0)	37 (80)	<0.01 ^†^
Scores				
APACHE II	9.0 (5.4–11.6)	11.0 (8.4–14.6)	15.0 (10.0–18.3)	<0.01 *
ROX on admission	6.4 (5.5–7.7)	4.3 (3.5–5.8)	4.1 (3.7–5.0)	<0.01 *
ROX on day 2	7.4 (5.3–9.2)	4.9 (4.1–5.8)	4.4 (3.6–5.7)	<0.01 *
ROX on day 3	7.8 (6.2–9.9)	5.6 (4.2–7.1)	4.2 (3.3–5.8)	<0.01 *

^†^ Pearson. * Wilcoxon. PH: acidity/alkalinity; PCO_2_: partial pressure of carbon dioxide; PaO_2_: partial pressure of oxygen; P/F: arterial oxygen partial pressure to fractional inspired oxygen; AST: aspartate transaminase; ALT: alanine aminotransferase; MODS: Multiple Organ Dysfunction Syndrome.

**Table 2:** Multinomial logistic regression of the group’s late vs. early and non- vs. early responders.

	Predictor	Estimate	SE	Z	*p*	Odds Ratio	95% Confidence Interval	
							Lower	Upper
Late vs. Early	Intercept	4.07379	2.0088	2.028	0.043	58.779	1.1463	3013.9
	Age	−0.00743	0.0301	−0.247	0.805	0.993	0.9357	1.053
	Sex: Female–Male	−1.35952	0.9257	−1.469	0.142	0.257	0.0418	1.576
	Mode of Intervention	−0.09002	0.7008	−0.128	0.898	0.914	0.2314	3.609
	ROX on admission	−0.75948	0.2373	−3.2	0.001	0.468	0.2939	0.745
	ROX on day 2	−0.512	0.191	−2.67	0.008	0.599	0.412	0.872
	ROX on day 3	−0.594	0.221	−2.69	0.007	0.552	0.358	0.851
	APACHE II	0.08962	0.0899	0.997	0.319	1.094	0.9171	1.304
	Sepsis	0.0719	1.109	0.0648	0.948	1.075	0.1222	9.452
	MODS	−0.3049	1.167	−0.261	0.794	0.737	0.0749	7.258
	Acute Kidney Injury	1.7603	1.197	1.471	0.141	5.814	0.5569	60.70
	Diabetes Mellitus	−0.00741	0.532	−0.0139	0.989	0.993	0.3496	2.82
	Hypertension	0.30225	0.531	0.5691	0.569	1.353	0.4777	3.83
	Chronic Cardiac disease	−0.22465	0.829	−0.271	0.786	0.799	0.1574	4.05
	Chronic Kidney disease	−0.22582	0.836	−0.270	0.787	0.798	0.155	4.11
	Chronic respiratory disease	0.79948	1.258	0.6355	0.525	2.224	0.189	26.18
Non- vs. Early	Intercept	2.26483	2.1752	1.041	0.298	9.629	0.1355	684.11
	Age	0.01381	0.0328	0.422	0.673	1.014	0.9508	1.081
	Sex: Female–Male	−1.96438	1.0491	−1.872	0.061	0.14	0.0179	1.096
	Mode of Intervention	0.71202	0.7939	0.897	0.37	2.038	0.43	9.661
	ROX on admission	−0.94045	0.2701	−3.482	<0.001	0.39	0.23	0.663
	ROX on day 2	−0.751	0.222	−3.39	<0.001	0.472	0.306	0.729
	ROX on day 3	−0.69	0.251	−2.75	0.006	0.502	0.307	0.82
	APACHE II	0.195	0.0498	3.92	<0.001	1.216	1.1024	1.34
	Sepsis	2.1241	0.914	2.3248	0.02	8.365	1.3956	50.14
	MODS	1.3667	1.009	1.3543	0.176	3.922	0.5427	28.35
	Acute Kidney Injury	1.7162	1.113	1.5421	0.123	5.563	0.6281	49.28
	Diabetes Mellitus	−0.62774	0.51	−1.2319	0.218	0.534	0.1966	1.45
	Hypertension	0.27071	0.51	0.5311	0.595	1.311	0.4827	3.56
	Chronic Cardiac disease	1.34248	0.672	1.997	0.046	3.829	1.0252	14.3
	Chronic Kidney disease	−1.22034	0.956	−1.2765	0.202	0.295	0.0453	1.92
	Chronic respiratory disease	0.73604	1.206	0.6101	0.542	2.088	0.1962	22.21

ROX: Ratio of Oxygen Saturation; APACHE: Acute physiology and chronic health evaluation; MODS: Multiple Organ Dysfunction Syndrome.

**Table 3:** Cut-off values for APACHE II; ROX indices for early vs. late responders.

	Cut Point	Sensitivity (%)	Specificity (%)	PPV (%)	NPV (%)	AUC
APACHE II on admission	14	84.62%	68.35%	56.90%	90%	0.847
ROX on admission	5.2	79.49%	74.68%	60.78%	88.06%	0.843
ROX on day 2	5.8	89.66%	67.31%	60.47%	92.11%	0.836
ROX on day 3	5.3	75.76%	73.53%	58.14%	86.21%	0.798

PPV: Positive Predictive Values; NPV: Negative Predictive Values; APACHE: Acute physiology and chronic health evaluation; ROX: Ratio of Oxygen Saturation.

**Table 4:** Cut-off values for APACHE II; ROX indices for responders **vs.** non-responders.

	Cut Point	Sensitivity (%)	Specificity (%)	PPV (%)	NPV (%)	Youden’s Index	AUC
APACHE II on admission	14	65.28%	76.09%	81.03%	58.33%	0.414	0.751
ROX on admission	4	73.21%	72%	85.42%	54.55%	0.452	0.759
ROX on day 2	4.6	61.11%	73.91%	78.57%	54.84%	0.35	0.734
ROX on day 3	5.3	74.60%	65.79%	78.33%	60.98%	0.404	0.745

PPV: Positive Predictive Values; NPV: Negative Predictive Values; APACHE: Acute physiology and chronic health evaluation; ROX: Ratio of Oxygen Saturation.

**Table 5:** Hazard ratio reflecting intubation risk was calculated using Multivariate Cox regression analysis.

	HR (Univariable)	HR (Multivariable)
Age	1.02 (0.99–1.05)	0.95 (0.84–1.06)
Sex (ref: Male)	0.55 (0.22–1.38)	0.28 (0.02–2.67)
ROX on admission	0.45 (0.32–0.60) ***	0.29 (0.13–0.52) ***
ROX on day 2	0.66 (0.47–0.92) *	0.55 (0.34–0.89) *
ROX on day 3	0.85 (0.61–0.92) *	0.83 (0.68–1.02)
APACHE II on admission	1.11 (1.03–1.19) **	1.12 (1.03–1.21) *
Hypertension	1.01 (1.00–1.03)	1.01 (0.98–1.04)
Chronic Cardiac disease	1.03 (1.01–1.06) **	1.09 (1.02–1.21) *
Chronic Kidney disease	1.16 (0.93–1.64)	1.83 (0.75–43.16)
Chronic respiratory disease	1.14 (0.51–2.48)	0.90 (0.13–6.16)
Complications		
Sepsis	8.56 (2.35–55.26) **	5.87 (1.27–45.18,) *
MODS	10.57 (3.44–46.37) ***	7.89 (2.12–40.58) **
Acute Kidney Injury	6.96 (2.25–30.63) **	5.80 (1.63–28.22) *

* = *p* < 0.05, ** = *p* < 0.01, *** = *p* < 0.001; HR: Hazard Ratio; ROX: Ratio of Oxygen Saturation; APACHE: Acute physiology and chronic health evaluation; MODS: Multiple Organ Dysfunction Syndrome.

## Data Availability

All data generated or analyzed during this study are included in this published article and are available from the corresponding author upon reasonable request.

## Abbreviations

**Abbreviation/Acronym**	**Definition/Full-Form**
AKI	Acute Kidney Injury
ALT	Alanine Aminotransferase
APACHE II	Acute Physiology and Chronic Health Evaluation
ARDS	Acute Respiratory Distress
AST	Aspartate Transaminase
AUC	Area Under the Curve
CI	Confidence Interval
COVID-19	Coronavirus Disease 2019
CPAP	Continuous Positive Airway Pressure
ECMO	Extracorporeal Membrane Oxygenation
HFNO	High-Flow Nasal Oxygenation
HR	Hazard Ratio
ICU	Intensive Care Unit
ISCCM	Indian Society of Critical Care Medicine
LMIC	Low- and Middle-Income Countries
MODS	Multi-Organ Dysfunction Syndrome
MOHFW	Ministry Of Health and Family Welfare
NIV	Non-Invasive Ventilation
NLR	Neutrophil-to-Lymphocyte Ratio
NPV	Negative Predictive Values
OR	Odds Ratio
PaO_2_	Partial Pressure of Oxygen
PaO_2_/FiO_2_	Ratio of Partial Pressure of Arterial Oxygen and Fraction of Inspired Oxygen
PCO_2_	Partial Pressure of Carbon Dioxide
PH	Acidity/Alkalinity
PPV	Positive Predictive Values
ROC	Receiver Operating Characteristic
ROX	Ratio of Oxygen Saturation
RTPCR	Reverse Transcription Polymerase Chain Reaction
RR	Respiratory Rate
SARS-CoV-2	Severe Acute Respiratory Syndrome Coronavirus 2
SEN	Sensitivity
SOFA	Sequential Organ Failure Assessment
SAPS	Simplified Acute Physiology Score
SPE	Specificity
SPO_2_/FIO_2_	Ratio of Oxygen Saturation to The Fraction of Inspired Oxygen
WBC	White Blood Count
